# Current Limitations of Murine Models in Oncology for Ascorbate Research

**DOI:** 10.3389/fonc.2014.00282

**Published:** 2014-10-14

**Authors:** Elizabeth J. Campbell, Gabi U. Dachs

**Affiliations:** ^1^Mackenzie Cancer Research Group, Department of Pathology, University of Otago, Christchurch, New Zealand

**Keywords:** vitamin C, xenograft, gulonolactone oxidase, hypoxia-inducible factor

## Abstract

The role of vitamin C (ascorbate) in cancer prevention, tumor growth, and treatment is of intense public interest. Clinical trial data have been sparse, contradictory, and highly controversial, and robust pre-clinical data are required for progress. This paper reviews pre-clinical models and their limitations with respect to ascorbate research. Most studies have utilized animals able to synthesize ascorbate and thus are not ideal models of the human condition. More recently, genetically modified mouse models have become available; yet, all studies compared healthy and scorbutic mice. The majority of investigations to date concluded that increased ascorbate led to decreased tumor growth, but data on mechanisms and doses are inconclusive. Clinically relevant animal studies are still required to convince a generally sceptical medical audience of the potential worth of ascorbate as an adjunct to therapy.

## Introduction

Vitamin C (ascorbate) is a small, water soluble molecule derived from glucose. Although most animals synthesize ascorbate in their liver or kidneys, it has become an essential vitamin for human beings due to mutations in the terminal enzyme of its biosynthesis pathway, l-gulonolactone oxidase (Gulo) (1, 2). Therefore, human beings, together with other primates, guinea pigs, and some fruit bats, require daily dietary ascorbate to maintain their health and prevent their tissues becoming ascorbate deficient (scorbutic). Although the extreme ascorbate deficiency condition, scurvy, is rare, large proportions of the population are at risk of chronic deficiency (3).

There are a myriad of roles attributed to ascorbate, with some potentially important in cancer risk and progression, but most data are derived from either cell-free or cell culture systems. Ascorbate is a potent antioxidant believed to protect immune cells against intracellular reactive oxygen species formed during the inflammatory response (4). Ascorbate supports both the innate and adaptive immune functions, inhibits excessive activation of the immune system, and maintains natural neutrophil apoptosis, thus preventing tissue damage (5). It supports immune cell activity, especially phagocytosis and T-cell function (4).

Ascorbate is a vital co-factor for a family of enzymes, the non-heme and 2-oxoglutarate-dependent dioxygenases, which control important cellular processes including hormone synthesis, protein structure, epigenetic changes, and response to low oxygen (hypoxia) (6–10). Hypoxia is a characteristic of solid tumors, and the extent of tumor hypoxia has been associated with more aggressive cancers and poor patient outcome (11). Hypoxia-inducible factor 1 (HIF-1) is the master regulator of cellular response to hypoxia, and HIF-1 levels and activity are under control of hydroxylase enzymes that require ascorbate as a co-factor (9). Ascorbate is essential for the development and maintenance of connective tissues and wound healing, via the synthesis of collagen. Collagen fibers form part of the extracellular matrix, a vital component of solid tumors. In cell culture studies, collagen increased directional migration of tumor cells, and introduction of ascorbate into the media reduced this metastatic phenotype (12).

The functional role of ascorbate in cancer biology is complex and remains unclear. Therefore, relevant animal models are crucial for investigating its role during tumor growth and cancer therapy. In contrast to cell culture systems, these *in vivo* models provide a complex microenvironment, which contains the appropriate physiological, immunological, and biomechanical components to model heterogeneous tumor growth. This review will give an overview of published data in animal models where the effect of ascorbate on cancer was investigated. We have divided animal models into two broad categories: ascorbate-synthesizing or ascorbate-dependent models.

## Ascorbate-Synthesizing Animals

Virtually all laboratory animals, including inbred wild type mice and immune-deficient murine models, can synthesize their own ascorbate.

### Wild type mice with murine tumors

Inbred wild type mice (e.g., C57BL/6, BALB/c, and C3H) have been used for >50 years to investigate tumor growth and treatment response. These mice have a fully competent immune response, which allows the study of the role of the immune system during therapy, but require mouse-strain specific murine tumor models (syngeneic tumors). A pharmacokinetic study in C57BL/6 mice following either IV or IP injection (1 g/kg) of ascorbate showed that plasma ascorbate can reach millimolar levels (13). Although IV administration resulted in an immediate increase in plasma concentrations (>20 mM), 1 h after administration there was little difference between IV and IP plasma ascorbate levels (~6 mM). Using BALB/c mice, Yeom et al. (14) showed that high-dose ascorbate injections (30 mg/mouse ~ 1 g/kg) inhibited sarcoma 180 tumor growth, and, dependent on time of administration, could play a preventative or therapeutic role through reduction of angiogenesis (14). Similarly, continuous high-dose IP injections (4 g/kg) produced a reduction in hormone-refractory prostate tumor volume and a decrease in metastatic lesions of over 50% (15). Furthermore, tumor growth of Lewis lung carcinomas and metastatic spread were reduced using a combination of IP vitamin C and vitamin K (16).

The maximum plasma levels achieved following injection with high-dose ascorbate in patients are about 100-fold higher than those achievable via dietary intake (17). Similarly, oral dosing in mice (6 g/L) did not reach pharmacological plasma levels (13). Combined treatment of ascorbate with vitamin K at dietary levels increased murine lifespan by 45% (18) and reduced metastasis in a liver cancer model (19). However, as ascorbate was not tested separately, its contribution is unclear.

From these reports, there appears to be an anti-tumor effect at high-dose ascorbate in wild type mice bearing syngeneic tumors.

### Immune-compromised mice with human tumor xenografts

The nude (Nu/Nu) mouse (20, 21) has been used in oncology for decades to characterize the growth, spread, and drug responses of human tumors (xenografts). This mouse strain has a mutation on chromosome 11 (Foxn1nu-), resulting in an absent thymus and greatly reduced number of T-cells, yet is able to mount a limited immune response (22–24). Human pancreatic tumor xenografts in nude mice treated with pharmacological levels of ascorbate (4 g/kg daily) were significantly smaller than untreated controls (7) and, using the same dosage, tumor volume of human ovarian, pancreatic, and glioblastoma models was significantly reduced (>40%) (25, 26). Similarly, daily IP injections of lower doses of ascorbate (0.15 g/kg) were associated with a reduction in tumor growth of human colorectal cancer xenografts (WiDr) (27), although this study was limited by small numbers per treatment group (*n* = 5). Human erythromyeloid leukemia tumors (K562), grown in nude mice, showed a 60% reduction in size compared to controls 4 weeks after a single IP injection of a combination of ascorbate/menadione (1 g/kg and 10 mg/kg, respectively) (28).

Severe combined immune deficiency (SCID) mice carry a mutation on chromosome 16 (DNA repair enzyme Prkdc^−^) and have lost the ability to produce B- and T-cells. However, these mice still contain low number of leukocytes and produce normal numbers of natural killer cells (29). Importantly, this mouse strain is unable to fight infections or reject tumors, and is, therefore, used widely in drug development studies using human tumor xenografts. When SCID mice were supplemented with ascorbate (5 g/L) in their drinking water, growth of human lymphoma xenografts was reduced, and this was attributed to an associated reduction in the transcription factor HIF-1 (30). In contrast, a study using prostate xenografts grown in SCID mice showed that PO (40 mg/kg or 500 mg/kg) administration of ascorbate was not sufficient to reduce tumor growth (31). Pharmacokinetic analysis conducted in this study indicated that plasma levels were elevated and maintained for at least 6 h (31), which is different to previous reports (13).

In summary, data from immune-compromised mice indicate an anti-tumor effect with high-dose ascorbate, dependent on dose and route of administration (Table 1).

**Table 1:** **Doses, routes, and schedules of pharmacological ascorbate administration as a single agent and tumor response**.

Model	Cancer cell (concentration)	Syngeneic or xenograft	Implantation	Tumor volume when treatment started	[VC]	Schedule	Administration	Ascorbate measurement	Tumor response	Reference
Ncr-nu/nu	PAN-02 (10^6^)	Xenograft	SC	30–40 mm^3^	4 g/kg	Daily; until EP	IP	No	Yes	(25)
Ncr-nu/nu	9L (2 × 10^6^)	Xenograft	SC	25–50 mm^3^	4 g/kg	Daily; until EP	IP	No	Yes	(26)
	Ovcar5 (5 × 10^6^)	Xenograft	
	Pan02 (1 × 10^6^)	Xenograft	
Ncr-nu/nu	MIA PaCa-2 (2 × 10^6^)	Xenograft	SC	3–4 mm^3^	4 g/kg	Twice daily; 14 days	IP	No	Yes	(7)
BALB/c nude	WiDr (8 × 10^6^)	Xenograft	SC	300–500 mm^3^	0.15 g/kg	Daily; 12 days	IP	No	Yes	(27)
SCID	P493-6 (3 × 10^6^)	Xenograft	SC	7 days BI + AI	5 g/L	Throughout	PO	No	Yes	(30)
SCID	EHMES-10 (5 × 10^6^)	Xenograft	SC	50 mm^3^	1 M solution	Single infusion	IV	No	Yes	(32)
BD2F mice	L1210 ascites (10^5^)	Syngeneic	IP	1 day AI	2 g/kg	Daily; until EP	IP	No	No	(33)
BALB/C	CT-26 (10^6^)	Syngeneic	IP	From outset	1.5 g/kg	Every 3 days	IP	No	Yes	(34)
NMRI mice	TLT (10^6^)	Syngeneic	IM	3 days AI	1 g/kg	Daily; 27 days	IV	Plasma (for PK study)	Yes	(13)
Lobund-Wistar rats	PAIII (10^6^)	Syngeneic	SC	10 days AI	4 g/kg	Daily; 30 days	IP	No	Yes	(15)

*PO, per os; IV, intravenous; IP, intraperitoneal; SC, subcutaneous; IM, intramuscular; AI, after implantation; BI, before implantation; EP, endpoint; DHA, dehydroascorbic acid*.

### Carcinogen-induced tumors in ascorbate-synthesizing animals

The potential role of ascorbate in the prevention of carcinogenesis has been investigated for many years, and recent studies are summarized here. The effect of dietary ascorbate on oestrogen-induced breast carcinogenesis was investigated in August Copenhagen Irish (ACI) rats (35). Ascorbate attenuated oestrogen-induced alterations in oxidative stress markers and enzyme activity, and reduced incidence of neoplastic lesions. In contrast, in some strains of rats, ascorbate increased carcinogen-induced bladder carcinoma (*N*-butyl-*N*-(4-hydroxybutyl)-nitrosamine), but in others it made no difference (36, 37). Similarly, in SKH-1 hairless mice exposed to genotoxic UV light, dietary ascorbate increased pre-cancerous and malignant skin lesions (38). The impact of ascorbate on carcinogenesis, therefore, remains unresolved.

In summary, few studies measured plasma levels and none measured tumor ascorbate levels, which is a vital starting point for mechanistic studies. Studies were difficult to compare, due to different doses and administration routes of ascorbate (Table 1). Most studies investigated high-dose ascorbate, and no clear molecular mechanisms were reported.

## Ascorbate-Dependent Animals

A number of early studies investigated ascorbate levels and tumor progression in the naturally deficient rodent, the guinea pig (39–41). In guinea pigs, ascorbate was reportedly preferentially taken up in tumor tissue (Dael and Biltris sarcoma) following IV injection of 50 mg ascorbate (42). Intermittent scurvy increased the frequency of carcinogen-induced tumors compared to healthy guinea pigs (43). In contrast, another study showed that increased ascorbate supplementation was associated with more rapidly growing tumors (41). In this study, guinea pigs, maintained on a scorbutic diet (0.3 mg/kg/day), showed increased tumor regression compared to animals maintained on higher doses (10 mg/kg/day or 1 g/kg/day), suggesting that tumors required ascorbate for growth. More recently, injection of ascorbate (500 mg/kg/day SC) or oral supplementation were shown to inhibited tumor growth in guinea pigs (44). Tumor growth was inversely correlated with tumor ascorbate levels, and when tumor ascorbate exceeded 1 mM, tumor growth was inhibited in >50% of animals. Thus, conflicts in the guinea pig data remain unresolved.

Improvements in genetic modifications have led to the production of a range of specific mouse models to study the role of ascorbate (Table 2). These genetically modified models contain deficiencies in ascorbate transporters and deficiencies in key points of the biosynthetic pathway.

**Table 2:** **Current ascorbate deficient murine models**.

Model	Description	Model type	Reference
GULO^−/−^	Deletions in the exons 3 and 4 rendering the GULO gene inactive.	GM	(45)
SFX mouse	Deletions in all 12 exons in the GULO gene.	Spontaneous	(46, 47)
SVCT1^–/–^	Exons 1–12 of the *Slc23a1* gene replaced by *NeoR*. SLC23A1^–^ shown to have significant reductions in renal reabsorption.	GM	(48)
SVCT2^–/–^	Deletion of 592 nucleotides causing mRNA frameshift. Deficiency of the transporter is lethal in neonates.	GM	(49)
GRKO/ARKO^−/−^	Double KO model in aldehyde reductase and aldose reductase. 95% reduction in ascorbate synthesis.	GM	(2)

*GULO, gulonolactone oxidase; KO, knockout; GM, genetically modified; SVCT, sodium-dependant vitamin C transporter*.

### Mice deficient in gulonolactone oxidase

Gulo^–/–^ mice are genetically modified C57BL/6 mice, which contain deletions in exons 3 and 4 of l-*gulonolactone oxidase*, rendering the enzyme inactive, and resulting in animals that require daily ascorbate supplementation, similar to human beings (45). As these animals have fully functional immune systems, they grow only syngeneic murine tumors.

Supplemented Gulo^–/–^ mice (500 ppm l-ascorbyl-2- polyphosphate with 150 mg/L ascorbate in drinking water) implanted with either 4TI breast tumors or B16F0 melanoma cells, showed reduced tumor growth and metastases compared with un-supplemented mice (50). Un-supplemented mice had ascorbate completely withdrawn for 4 weeks prior to tumor implantation, which has previously been shown to result in undetectable levels of ascorbate in plasma and most tissues, a significant reduction in mouse weight, and classical symptoms of scurvy and severe ill health (51). Using an ovarian tumor model, Kim et al. (52) showed that supplementation with oral ascorbate (1 g/L) reduced tumor growth and prolonged the onset of disease progression in tumor bearing Gulo^–/–^ mice, again in comparison to un-supplemented mice (52). Conflicting data were reported by Telang et al. (53), who showed that ascorbate depletion resulted in restricted tumor growth of Lewis lung carcinoma via restriction of angiogenesis, suggesting that a reduction of ascorbate may be a therapeutic approach (53).

Ascorbate uptake has been investigated in Gulo^–/–^ mice (54, 55). Depletion of ascorbate led to enhanced expression of the SVCT transporters in the liver, small intestine, and cerebellum, with an associated increase in ascorbate uptake in isolated hepatocytes from Gulo^–/–^ mice compared with wild type mice (54, 55).

“Spontaneous fracture” or “*Sfx*” mice are an inbred strain in a BALB/c genomic background. These mice were first described to contain an autosomaly recessive mutation, in which litter mates were phenotypically identical until ~6 weeks old when body mass, bone, and metabolic function decreased (47). The *sfx* gene has since been located to chromosome 14 and it is now known to contain the deletion of all 12 exons in the gene for l-gulonolactone oxidase (46). The Sfx mice require daily supplementation with adequate ascorbate to prevent scurvy, similar to the C57BL/6 Gulo^–/–^ mice. An inverse relationship between the level of ascorbate supplementation and tumor weight of B16F0 tumors was demonstrated in this model (56). In contrast, Parsons et al. (57) found no difference between ascorbate depleted mice and controls in respect to mammary tumor growth (57).

All above studies compared fully supplemented Gulo^–/–^ mice with severely scorbutic animals, which is likely to confound conclusions regarding the impact of ascorbate on cancer.

### Mice deficient in ascorbate transporters

Ascorbate is transported by two mechanisms, either by direct transport (sodium-dependent vitamin C transporters SVCT1 and 2), or by the uptake of dehydroascorbic acid (DHA) via glucose transporters, and subsequent intracellular reduction to ascorbate. SVCT1 is expressed on epithelial tissues such as intestine and kidney, where it contributes to the supply and maintenance of whole-body ascorbate levels, whereas expression of SVCT2 is more widespread, and governs the cellular concentration of ascorbate (58). A recent study in BALB/C mice bearing breast cancer xenografts analyzed the effect *in vivo* of manipulating tumor SVCT2 levels (59). It demonstrated that an increase of SVCT2 levels was associated with increased tumor growth delay following daily IP ascorbate injections (1 g/kg), and a reduction of SVCT2 was associated with a reduction in tumor response (59). These results indicate that intracellular ascorbate concentrations play a major role in tumor response to high-dose vitamin C treatment.

Although SVCT-knockout mice have been created (48, 49), they have not yet been used in cancer research. There is evidence that mice and rats have reduced (SVCT-dependent) ascorbate transport compared to human beings and guinea pigs (51, 60, 61). This will affect studies on ascorbate transport and metabolism in those animals, but should not impact on research into cancer if plasma and tissue levels are monitored.

### Mice deficient in aldehyde reductase and aldose reductase

Recently, the *aldehyde reductase (GR)* and *aldose reductase (AR)* double knockout (GRKO/ARKO) mouse has been established on a C57BL/6 background (2). This model silences key enzymes in the ascorbate biosynthetic pathway, leading to a 95% reduction in liver synthesis of ascorbate. Similar to Gulo^–/–^ mice, these animals will become scorbutic without supplementation. This model has not yet been used in cancer research.

## Potential Mechanisms in Cancer

There is sparse evidence for the role of ascorbate in the tumor microenvironment, and factors likely to influence tumor response include species, tumor type, size at treatment, whether tumors were grown subcutaneously or orthotopically, ascorbate transporter status, and vascularization, which have all not been rigorously investigated. With the exception of early data in guinea pigs (42), few studies have reported plasma levels and none have investigated tumor ascorbate levels. As tumor ascorbate following intervention in neither human beings nor mice has ever been measured, the optimal dose for maximum tumor concentrations is unknown. A fundamental issue of ascorbate and cancer therapy is the lack of robust data for ascorbate’s roles *in vivo*. Pharmacological versus physiological levels of ascorbate will have significantly different effects.

At pharmacological levels, ascorbate may mediate an anti-tumor effect via apoptosis (32, 62), ATP depletion (26, 63), or autophagy (7). High doses of ascorbate are believed to act as prodrug in tissue culture to generate H_2_O_2_ and induce cell kill (64). Chen and colleagues (26, 63), have shown that pharmacological levels of ascorbate can induce a pro-oxidant state through H_2_O_2_ production, which may lead to ATP depletion. Du et al. (7) have shown that increased ascorbate concentrations stimulated an apoptotic response and, at pharmacological levels of ascorbate, autophagy, consistent with previously published work (65). However, these conclusions were drawn from *in vitro* studies, and although *in vivo* work was carried out using the same cell line (MIA Pac-2), the mechanism was inferred and no biomarkers of oxidation were measured.

Pharmacological doses have been used without increased liver toxicity in mice (14, 26), and these doses have been shown to potentiate the cytotoxtic effect of some chemotherapeutic drugs in mice (28, 66, 67), whilst offering cardioprotection from the cytotoxic side effects (68–70). One notable exception was reported by Heaney et al. (71), who demonstrated a reduction in efficacy of a range of chemotherapeutic agents when given in combination with DHA (71). DHA, the oxidized form of ascorbate, circulates at low levels *in vivo* of about 2–10 μM compared with 50–70 μM of ascorbate, and is thus unlikely to play a major role (72).

At physiological levels, the role of ascorbate in the control of HIF-1 is the most investigated and plausible pathway to date. *In vitro* cell culture and enzyme studies have provided robust mechanistic data of the role of ascorbate as co-factor for the oxygen-sensing HIF hydroxylases (5, 73–75), although this has been refuted (76). In all other studies, increasing ascorbate levels were associated with reduced HIF-1 levels *in vitro* (10) and *in vivo* (30). In addition, levels of two targets of HIF-1, the metallo-enzymes MMP-2 and 9, were significantly reduced in the presence of increased ascorbate (16).

However, all current *in vivo* data are based either on high-dose treatment in ascorbate-synthesizing mice (25, 26, 30) or dietary interventions in ascorbate-dependent animals, but comparing healthy and scorbutic mice (50, 53), and may, therefore, not be relevant for human beings. As tumor levels have not systematically been measured following high-dose ascorbate treatment in these mouse studies, it is unclear whether pharmacological, toxic levels were achieved, thus accounting for an anti-tumor effect via the proposed production of H_2_O_2_. Or, alternatively, whether these high doses were required to overcome the poor vascularization characteristic of solid tumors, thus achieving physiological concentrations and regulating factors such as HIF-1 via ascorbate’s co-factor role. Levels and function of ascorbate transporters, SVCT1 and SVCT2, in tumor tissue have only recently started to be investigated (59), and differences between species will affect tissue levels (61). The other potential roles of ascorbate during cancer growth, such as epigenetic changes and immune function, have also not been investigated.

## Conclusion

Ascorbate could be an inexpensive addition to standard cancer treatment if proven effective. Current animal models have provided evidence of, and some mechanistic insight into, the anti-tumor effects of ascorbate. However, the limited use of suitable ascorbate dependent, yet non-scorbutic, models has limited the strength of the evidence. In addition to using ascorbate-synthesizing animals, many studies are weakened by using a wide range of doses and administration routes that are inconsistent among studies, and seldom measure circulating or tissue ascorbate levels. The role of species-specific gene regulation of ascorbate synthesis and transport may further complicate translation from animals into patients. Hence, carefully conducted, clinically relevant, pre-clinical studies are still required to convince a generally sceptical medical audience of the potential worth of ascorbate as an adjunct to therapy.

## Conflict of Interest Statement

The authors declare that the research was conducted in the absence of any commercial or financial relationships that could be construed as a potential conflict of interest.

## Abbreviations

HIF-1, hypoxia-inducible factor 1; IP, intraperitoneal; IV, intra venous; PO, *per os* (by mouth); SC, subcutaneous.
